# Protecting impact of Jaft against carbendazim induced biochemical changes in male Wistar rats

**Published:** 2015

**Authors:** A Mirzaei, S Sepehri, H Sadeghi, A Alamdari

**Affiliations:** * Medicinal Plants Research Center, Yasuj University of Medical Sciences, Yasuj, Iran,; ** Department of Biotechnology, Fergusson College, Pune – 414001, Maharashtra, India

## Abstract

**Introduction:** Pesticides are a critical tool for crop protection and control of different pests and insects. The present research conducted to evaluate the protective role of Jaft extract against oxidative pressure, biochemical variations because of limited time giveaway to carbendazim in Wistar mice males.

Fresh fruits of *quercus brantii* were dried and the internal layer (Jaft) was collected for a hydroalcoholic extract by a maceration method at normal ambient condition. For the experimental study, twenty-four adult male rats (Wistar albino rats weighing 150-200 g) were randomized into 3 teams out of eight.

Team I subserved like a vehicle treated group, received corn oil additionally to their food, while the animals in the second team got 0.1 ml carbendazim (50mg/ kg in corn oil) via oral path for nine days. Rats in group III received Jaft (500 mg/ kg orally + in carbendazim for 9 days. Blood samples were obtained by heart puncture to determine alkaline phosphatase (ALP), blood urea nitrogen (BUN), creatinine, alanine (ALT) and aspartate aminotransferases (AST); by using auto-analyzer in serum.Kidneys and liver separated from rats and provided for series of biochemical parameters homogenization like GSH and MDA stages.

**Result:** The serum content of AST, ALT, ALP, BUN and creatinine were significantly elevated by in carbendazim treatment (group II) compared to the negative group (p<0.01).The liver enzymes operations, creatinine and BUN were significantly reduced in rats (p<0.05) when Jaft was received in a short period of time (group III). Hepatic and GSH and renal MDA stages in group (II) were clearly (p<0.05) enhanced and decreased consequently. The GSH and MDA stages content were significantly normalized in mice (p<0.05) when Jaft was received by group III.

**Conclusions:** According to the present data, Jaft can neutralize carbendazim contain pressure of oxidative and recover the abnormal pathological injuries in Wistar mice males.

## Introduction

Today, pesticides are an important tool for crop protection and control of different pests and insects. Carbendazim (methyl-2-benzimidazole carbamate) with systemic broad-spectrum is a fungicide agent that is comprehensively utilized in gardening and agricultural disease control program. In addition it is employed as preservative in paint, textile, paper, leather and fruit crop industry [**1**].

Carbendazim is an end product of benomyl, the most extensive ecological pollutant related to human and animal reproductive health. Carbendazim is a toxic substance according to the World Health Organization classification, which is broadly used as a fungicide agent [**2**]. It acts on tubulin via interferes in microtubule development and meiotic cell division [**2**].

Man may be exposed to carbendazim either through environmental contamination or through occupational exposures. Several diseases such as hypertension, eyes, nose and throat irritation and headache were reported due to occupational exposure [**3**,**4**]. Carbendazim and related metabolite benomyl were identified to cause toxicity of testicular via immature spermatids [**5**] and interference of microtubule association.

Also, in mammals exposure with carbendazim is associated with disturbances in performance of liver, reproduction and hematopoiesis procedure [**6**].

According to some research results, carbendazim can induce damages to thyroid, parathyroid , adernal glands and some hormone content in rats [**7**].

It was supposed that the Jaft extract may become a new substance in the near future, to control inflammatory disease and oxidative stress pathogenesis in man and animals. Hence, the present research was carried out to evaluate the protective effect of Jaft extract against oxidative stress and variation of biochemical issues due to short-term exposure to carbendazim in male Wistar rats.

## Methodology

**Preparation of extract**

Fresh fruits of *quercus brantii* were collected form Yasuj Iran. The fruits were dried and an internal layer of the fruit (Jaft) was collected. The extraction of Jaft conducted at ambient condition for 2 days by maceration method, and ethanol 70% as a solvent employed. The plant extract was filtered by using whatman No. 1 paper of filter and focused by rotary evaporator (BUCHI, Switzerland) at 400C. The raw essence stored in fridge for more investigation.

Twenty four Adult male Wistar albino rats weighing 150-200 g get of our rat colony.

The animals were maintained in a 12-hour light/ dark cycle, at 20°C ± 2°C, with the 50% ± 10% humidity. Animals were fed based on the standard rodent food pellets and natural water *ad libitum* for the entire test period. The experimental protocol was carried out based on the international guides about the animal’s correct care and application in experimental tests which confirmed via the local ethics committee. Animals were divided into three groups of eight each.

First team behaved like vehicle manned group, receiving corn oil additionally to their food, when in second team animals got 0.1 ml carbendazim (98.3% pure) (50mg/ kg in corn oil) orally for nine days. Rats in group III received Jaft (500 mg/ kg by oral route + carbendazim for 9 days. All the animals in all groups moved faster during night and scarified [**2**, **8**, **9**].

Blood samples were obtained by heart puncture under light ether anesthesia to specify the ALT, AST, ALP, creatinine, and BUN by using the auto-analyzer in serum. The serum centrifuged for 10 min at 3000 g. 

## Statistical analysis 

The statistical analysis was carried out by using the variance one-way analysis. The values were stated as average ± normal Deviation (SD). Amounts of p <0.05 are assumed as analytically obvious.

## Results

**Fig. 1:**
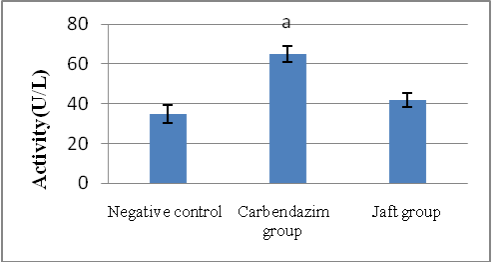
The impacts of Jaft extract on serum alanine aminotransferase (ALT )enzyme activity in Carbendazim induced biochemical changes

**Fig. 2:**
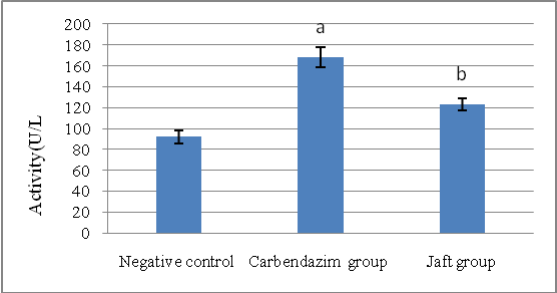
The effects of Jaft extract on serum aspartate aminotransferase (AST) enzyme activity in Carbendazim induced biochemical changes in male Wistar rats

**Fig. 3:**
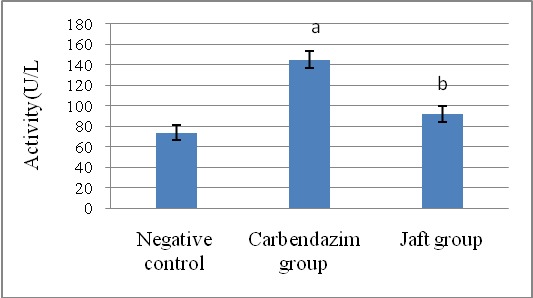
The effects of Jaft extract on serum ALP enzyme operation in Carbendazim induced biochemical changes in male Wistar rats

The serum activities of ALP, ALT, and AST were significantly elevated by in carbendazim treatment (group II) related to the negative team (p<0.01). The liver enzyme activities were significantly reduced in rats (p<0.05) when Jaft was received in a short time (group III) (**Fig. 1**-**3**).

**Fig. 4:**
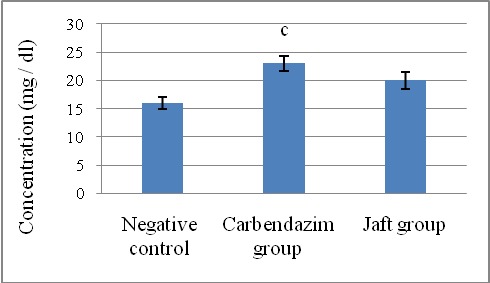
The effects of Jaft extract on serum blood urea nitrogen in Carbendazim induced biochemical changes in male Wistar rats

**Fig. 5:**
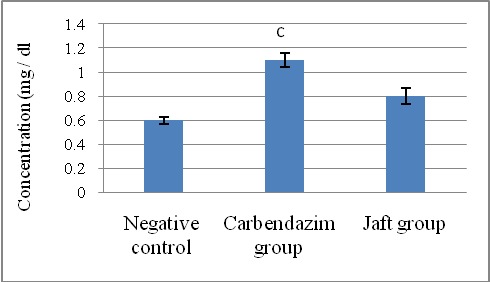
The effects of Jaft extract on serum creatinine in Carbendazim induced biochemical changes in male Wistar rats

Creatinine and BUN are clearly (p<0.05) raised because of carbendazim therapy (Second team) whilst their stages in the third team were relative to comparing to the values in the control group (**Fig. 4**,**5**).

As a lipid peroxidation marker in hepatic and renal tissues, MDA were significantly (p<0.05) increased after a short-duration showing to carbendazim (second team). The MDA contents were significantly reduced in rats (p<0.05) when Jaft was received in short time (third team) (**Table 1**).

In carbendazim showing (second team) glutathione (GSH) was clearly decreased (p<0.001) in the renal tissues and hepatic related to the negative control however, the height of the GSH content was reported in the Jaft treated rats (group III) (**Table 2**).

(I): Negative control received corn oil; (II): received carbendazim 50 mg/ kg for 9 days; (III): received carbendazim 50 mg/ kg + Jaft extract 500 mg/ kg treated for 9 days.

aStatistically significant difference versus negative group (P < 0. 01). Values are mean ± SD from 8 rats in each group

(I): Negative control received corn oil; (II): received carbendazim 50 mg/ kg for 9 days; (III): received carbendazim 50 mg/ kg + Jaft extract 500 mg/ kg treated for 9 days. Values are mean ± SD from 8 rats in each group.

a Analytically notable and clear distinct in comparison to negative one (P < 0. 01). 

b Analytically notable and clear distinct in comparison to Carbendazim one (P < 0. 05). 

(I): Negative control received corn oil; (II): received carbendazim 50 mg/ kg for 9 days; (III): received carbendazim 50 mg/ kg + Jaft extract 500 mg/ kg treated for 9 days. Values are mean ± SD from 8 rats in each group.

a Analytically clear distinct in comparison negative team (P < 0. 01).

b Analytically clear distinct in comparison Carbendazim team (P < 0. 05).

(I): Negative control received corn oil; (II): received carbendazim 50 mg/ kg for 9 days; (III): received carbendazim 50 mg/ kg + Jaft extract 500 mg/ kg treated for 9 days. Values are mean ± SD from 8 rats in each group

c Analytically clear distinct in comparison negative team (P < 0. 05). 

(I): Negative control received corn oil; (II): received carbendazim 50 mg/ kg for 9 days; (III): received carbendazim 50 mg/ kg + Jaft extract 500 mg/ kg treated for 9 days. Values are mean ± SD from 8 rats in each group

c Analytically clear distinct in comparison negative team (P < 0. 05). 

**Table 1:** The impacts of Jaft extract on hepatic GSH and MDA contents in Carbendazim induced biochemical changes in male Wistar rats

Teams	MDA	GSH
Negative control	61 ± 12.4	9.14 ± 1
Carbendazim group	105 ± 11.6	6.12 ± 0.57
Jaft group	88 ± 13b	7.4 ± 0.49 b

Jaft: internal layer of oak fruits, MDA: Malondialdehyde, GSH: reduced glutathione. Values are mean ± SD from 8 rats in each group

a Analytically clear distinct in comparison negative team (P < 0.001).

b Analytically clear distinct in comparison Carbendazim team (P < 0.05).

**Table 2:** The impacts of Jaft extract on GSH and renal MDA contents in Carbendazim induced biochemical changes in male Wistar rats

Groups	MDA	GSH
Negative control	61 ± 8.9	9.12 ± 1.1
Carbendazim group	98 ± 7.7 a	5.4 ± 0.44a
Jaft group	76 ± 9.1b	7.2 ± 0.49b

Jaft: oak fruits internal layer, MDA: Malondialdehyde, GSH: reduced glutathione. Values are mean ± SD from 8 rats in every team

Analytically notable and clear distinct in comparison to negative one (P < 0.001). 

Analytically notable and clear distinct in comparison to one Carbendazim (P < 0.05).

## Discussion 

In this investigation, a clear and notable enhance in ALP, AST, and ALT activities in Carbendazim exposure (group II) were reported and this finding was similar via the findings of many researchers [**2**].

Elevation of liver enzyme markers suggested early signs of hepatocytes injuries due to carbendazim exposure.

In the current study, a clear enhance in creatinine and BUN focuses stated because of the disturbance in renal function (p >0.05). Similarly, Selmanoglu et al. [**2**] observed increased levels of creatinine, cholesterol, and albumin in male rats treated with carbendazim.

Generally, the increase in creatinine content occurs with renal failure. The concomitant treatment of the Jaft extract was successful vice versa to the elevated levels of ALT, AST, ALP, BUN and creatinine, but the more efficacy might be possible with continued Jaft extract treatment [**2**].

The administration of carbendazim caused an elevation in lipid peroxidation content in blood which can be connected to the production of independent rebels. The present findings match via the findings of Eun Young and Ju-chan, Muthuviveganandavel et al., Saber et al., Saber and Somaya, whom stated that carbendazim induced hepatotoxicity [**2**]. 

According to Sakret al. [**10**], mancozeb fungicide in albino rats caused a significant reduces like an antioxidant enzyme in the tissue superoxide dismutase and an enhancement in lipid peroxidation [**11**]. 

The enhancement in lipid peroxidation indicates the oxidative pressure production, which is an imbalance between the independent radical’s generation and the body defense system [**12**]. In this research, lipid peroxidation levels are clearly and notably less in the Jaft extract treated groups compared to negative control, thus, the Jaft extract may exert antioxidant activities and protect the tissues from lipid peroxidation. Antioxidant activity of Jaft was reported in our laboratory.

The oxidative damage of carbendazim on blood was indicated in our study by the higher MDA stages; lipid peroxidation generation, together with lower content of GSH activity in blood treated with carbendazim related to natural control.

GSH is the critical non-protein sulfhydryl antioxidant in the cell. In the estimation of oxidative pressure, glutathione concentration is a good marker [**13**]. Similar to the current study, blood GSH concentration was significantly decreased that can be because of an increased utilization by Glutathione peroxidase [**14**]. 

Glutathione is a tripeptide, which is concentrated in erythrocytes. Glutathione has different functions including the cell cycle regulation and gene expression and play a part in xenobiotics and eicosanoides metabolism [**15**]. The increase in lipid peroxidation in carbendazim treated rats, beside the decrease in GSH concentration is suggestive of oxidative stress. This finding is parallel to many researchers [**2**]. Banks and Soliman showed that benomyl significantly increases the lipid peroxidation and reduced blood GSH in rats. 

## Conclusions 

In the present research, the increase of reduced GSH activity was reported in the Jaft treated rats. It could be that Jaft can diminish free radical and lipid peroxidation damages and improved the capacity of antioxidant enzymes. 
